# Predicting adverse outcomes in pregnant patients positive for SARS-CoV-2: a machine learning approach- a retrospective cohort study

**DOI:** 10.1186/s12884-023-05679-2

**Published:** 2023-08-02

**Authors:** Dylan Young, Bita Houshmand, Chunyi Christie Tan, Abirami Kirubarajan, Ashna Parbhakar, Jazleen Dada, Wendy Whittle, Mara L. Sobel, Luis M. Gomez, Mario Rüdiger, Ulrich Pecks, Peter Oppelt, Joel G. Ray, Sebastian R. Hobson, John W. Snelgrove, Rohan D’Souza, Rasha Kashef, Dafna Sussman

**Affiliations:** 1 Department of Electrical, Computer and Biomedical Engineering, Toronto Metropolitan University (formerly Ryerson University), 350 Victoria St, Toronto, ON M5B 0A1 Canada; 2 Institute for Biomedical Engineering, Science and Technology (iBEST), Toronto Metropolitan University & St. Michael’s Hospital, Toronto, Canada; 3 Keenan Research Centre for Biomedical Science, St. Michael’s Hospital, Toronto, Canada; 4 MD Program, Temerty Faculty of Medicine, University of Toronto, Toronto, Canada; 5 Department of Obstetrics and Gynecology, McMaster University, Hamilton, Canada; 6 Department of Obstetrics and Gynaecology, Faculty of Medicine, University of Toronto, Toronto, Canada; 7 Department of Obstetrics and Gynaecology, Mount Sinai Hospital, Toronto, Canada; 8 Department of Obstetrics and Gynecology, Division of Maternal-Fetal Medicine, INOVA Health System, Falls Church, VA USA; 9 Saxony Center for Feto-Neonatal Health, Medizinische Fakultät Der TU Dresden, Dresden, Germany; 10 Department of Obstetrics and Gynaecology, University Hospital of Schleswig-Holstein, Kiel, Germany; 11 Department for Gynecology, Obstetrics and Gynecological Endocrinology, Kepler University Hospital Linz, Johannes Kepler Universität Linz, Altenberger Strasse 69, 4040 Linz, Austria; 12 Department of Obstetrics and Gynaecology, St, Michael’s Hospital, Toronto, Canada; 13 Department of Obstetrics & Gynaecology and Health Research Methods Evidence and Impact, McMaster University, Hamilton, Canada

**Keywords:** Machine learning, Prognostication, Pregnancy, SARS-CoV-2, COVID-19

## Abstract

**Background:**

Pregnant people are particularly vulnerable to SARS-CoV-2 infection and to ensuing severe illness. Predicting adverse maternal and perinatal outcomes could aid clinicians in deciding on hospital admission and early initiation of treatment in affected individuals, streamlining the triaging processes.

**Methods:**

An international repository of 1501 SARS-CoV-2-positive cases in pregnancy was created, consisting of demographic variables, patient comorbidities, laboratory markers, respiratory parameters, and COVID-19-related symptoms. Data were filtered, preprocessed, and feature selection methods were used to obtain the optimal feature subset for training a variety of machine learning models to predict maternal or fetal/neonatal death or critical illness.

**Results:**

The Random Forest model demonstrated the best performance among the trained models, correctly identifying 83.3% of the high-risk patients and 92.5% of the low-risk patients, with an overall accuracy of 89.0%, an AUC of 0.90 (95% Confidence Interval 0.83 to 0.95), and a recall, precision, and F1 score of 0.85, 0.94, and 0.89, respectively. This was achieved using a feature subset of 25 features containing patient characteristics, symptoms, clinical signs, and laboratory markers. These included maternal BMI, gravidity, parity, existence of pre-existing conditions, nicotine exposure, anti-hypertensive medication administration, fetal malformations, antenatal corticosteroid administration, presence of dyspnea, sore throat, fever, fatigue, duration of symptom phase, existence of COVID-19-related pneumonia, need for maternal oxygen administration, disease-related inpatient treatment, and lab markers including sFLT-1/PlGF ratio, platelet count, and LDH.

**Conclusions:**

We present the first COVID-19 prognostication pipeline specifically for pregnant patients while utilizing a large SARS-CoV-2 in pregnancy data repository. Our model accurately identifies those at risk of severe illness or clinical deterioration, presenting a promising tool for advancing personalized medicine in pregnant patients with COVID-19.

**Supplementary Information:**

The online version contains supplementary material available at 10.1186/s12884-023-05679-2.

## Introduction

As of March 2023, Coronavirus Disease of 2019 (COVID-19) from Severe Acute Respiratory Syndrome Coronavirus 2 (SARS-CoV-2) infection has resultedin over 676,000,000 confirmed cases and 6,800,000 confirmed deaths worldwide [1]. Pregnant individuals are more likely to become severely ill compared to non-pregnant people [2–4]. Infection with SARS-CoV-2 in pregnancy can result in acute respiratory distress syndrome (ARDS), multiple organ failure, or maternal or fetal death [5]. Pregnant individuals are particularly vulnerable to respiratory viruses due to the physiologic and immunologic changes associated with pregnancy and higher oxygen demands related to fetal-placental circulation [5]. During the SARS-epidemic in 2003, approximately 50% of pregnant patients with SARS-CoV were admitted to an intensive care unit (ICU), 33% required mechanical ventilation, and 25% experienced fatal outcomes [6]. Comorbidities such as obesity, hypertension, pre-existing respiratory illness, and type 2 diabetes are known major risk factors for severe complications of COVID-19 during pregnancy [7, 8]. Other probable predictors in the general population include abnormal laboratory markers, radiological lung features, and respiratory parameters [7]. Despite these known risk factors, it remains challenging to predict the clinical course of pregnant patients with SARS-CoV-2, which can range from asymptomatic or mild disease to the most severe of outcomes [9, 10]. A reliable method for identifying individuals at-risk for detrimental severe outcomes would greatly benefit this vulnerable population by allowing clinicians to begin medical intervention early on in the disease course. As the number of global cases rises, access to large data repositories of COVID-19 cases in pregnant people has provided new opportunities for the development of Artificial Intelligence (AI) models.

AI algorithms use a variety of approaches to imitate human intelligence for automatic decision-making. Since the discovery of SARS-CoV-2, AI and in particular its subfield of machine learning (ML), has been used successfully to support clinical decision-making in COVID-19 cases [11–13]. In particular, deep learning methods, a subtype of ML, have found success in applying neural network architectures which vaguely mimic the brain by propagating information through a series of nodes which manipulate the information into useful forms [14]. The propagation process is systematically adjusted through a “training” stage where parameters of the network are optimized to improve performance of the network [14]. Using AI and a comprehensive SARS-CoV-2 in pregnancy data repository, we have developed a robust COVID-19 prognostication algorithm for pregnant patients with COVID-19. The purpose of this algorithm is to conduct higher-level risk-stratification upon obtaining a positive SARS-CoV-2 test such that individual maternal and fetal factors can be considered in clinical decisions for more efficient patient treatment pipelines. Our prognostication algorithm allows for effective triaging of this vulnerable population in resource-strained healthcare systems by determining whether a pregnant person is at risk of severe outcomes. Identifying low-risk individuals could facilitate outpatient management and virtual antenatal care without the need for in-person assessments and laboratory tests, thus decreasing the time and financial load on healthcare systems.

## Methods

### Study design

We developed and evaluated a novel prognosis-aid for early risk assessment of pregnant patients with SARS-CoV-2 based on retrospective international data from three countries (Canada, Austria, and Germany). The primary objective of our algorithm was to provide high-accuracy prediction of an adverse outcome for pregnant patients. The model was developed through a series of steps including preprocessing, feature selection, and algorithm creation. Multiple machine learning models were trained and compared to find the optimal model. This study was conducted in accordance with the Transparent Reporting of a Multivariable Prediction Model for Individual Prognosis or Diagnosis (TRIPOD) Statement [15].

### Study population and dataset

Two independent datasets of patients who tested positive for SARS-CoV-2 during pregnancy were used to create a single international data repository of 1501 pregnant patients: (a) The COVID-19 Related Obstetric and Neonatal Outcome Study (CRONOS) dataset from Germany and Austria established by the German Society of Perinatal Medicine (DGPM) consisting of 1402 patients and (b) The Mount Sinai Hospital (MSH) dataset from Canada consisting of 99 patients. The repository comprised multiple common variables, including demographics, comorbidities, symptoms, respiratory parameters, radiological results, and laboratory markers. These data points were documented either at the time of a positive SARS-CoV-2 test or at a time most recent to the positive test. To create a consistent dataset usable for ML, only features available at the time of COVID-19 testing were used to make predictions, and other values available from later points in the pregnancy were not used. Laboratory markers and symptoms would reflect the patients’ immediate health at the time of measurement.

**Fig. 1:**
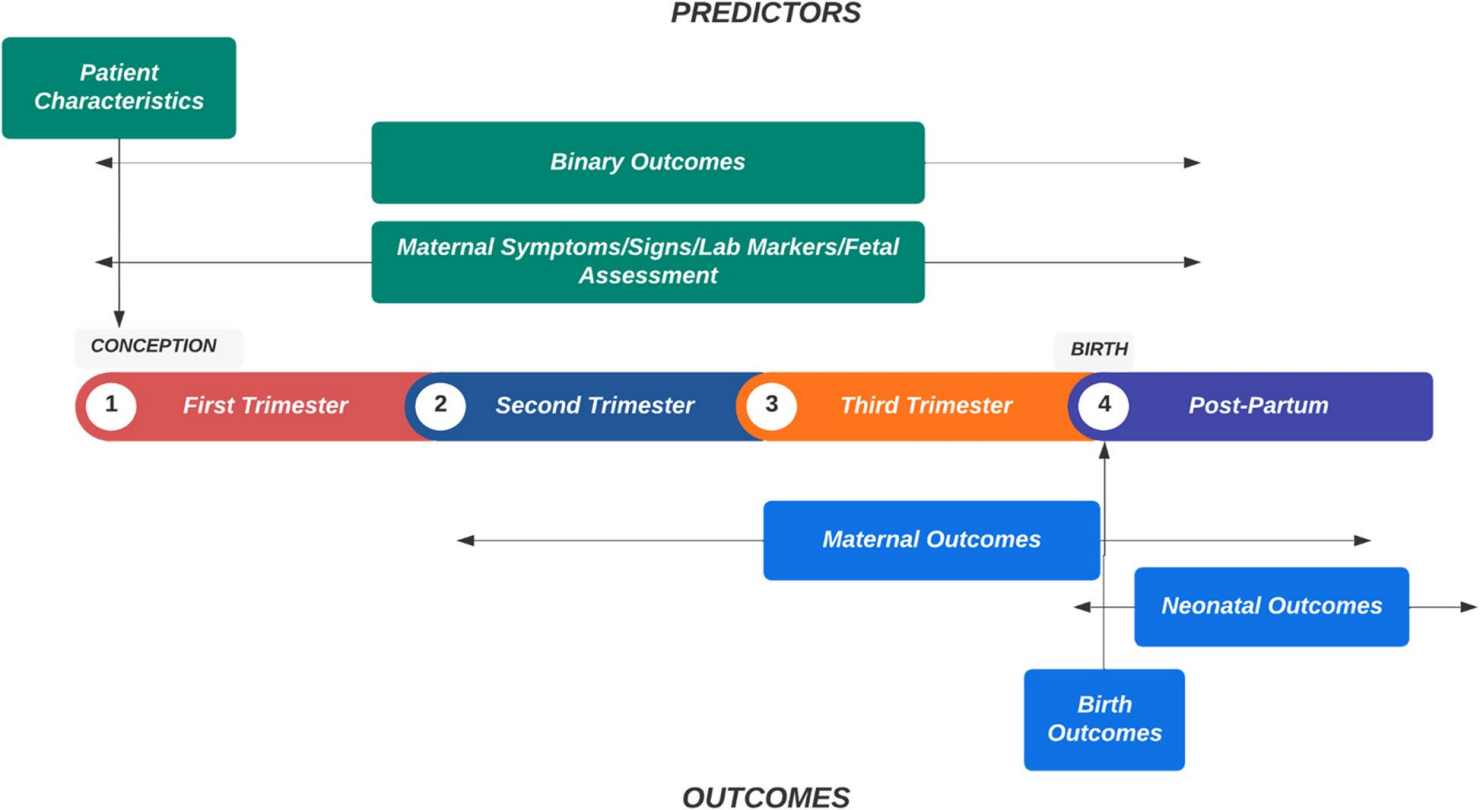
Timeline of predictors (features) and adverse pregnancy outcomes

Figure 1 describes the time at which the predictors (selected features) and outcomes occurred. Some predictors occur at a single time point in pregnancy, while others could change during pregnancy. Many predictors and outcomes could arise throughout the entire course of the pregnancy, making temporality an important concern. We will only use predictors available at the time of a positive test to study outcomes that haven’t occurred yet. For example, features in the category ‘patient characteristics’ are determined early in the pregnancy and are not modified during pregnancy (BMI, gravidity, parity, pre-existing comorbidities). These would be applicable to any stage of the pregnancy. Those included under ‘binary outcomes’ (Y/N), would be considered Yes if they occurred at any time prior to the positive test (smoking exposure, use of antihypertensives, diagnosis of fetal malformations on a mid-trimester ultrasound scan, administration of antenatal corticosteroids). The ‘values’ used for symptoms, signs, laboratory tests, and assessment of fetal wellbeing would be those recorded at the time of the positive test, disregarding any prior record of events. Finally, outcomes that will be predicted will only include events that were relevant to the SARS-CoV-2 infection. A study by Gold et al [16] demonstrated their proposed algorithms both for how patient symptoms collected at the time of COVID-19 diagnosis can be used to determine whether pregnant patients require inpatient management, and how clinical signs, laboratory markers, and radiological findings can help identify at-risk patients. Data collection methods such as these would be well-suited for the application of a pregnant patient prognostication model.

### Ethics

Ethics approval was obtained from Ryerson University (REB 2020–310, Aug 13, 2020) and MSH (20–0160-C, July 16, 2020) Research Ethics Boards. Data-use and collaboration agreements were established with CRONOS and MSH to facilitate the transfer of de-identified patient data to Ryerson University to build the repository. Ethics approval for the CRONOS dataset was obtained by the DGPM from the University Hospital of Schleswig–Holstein Campus Kiel (reference number: D 451/20, amended Feb 15, 2021, for this collaboration). The CRONOS Consortium holds individual ethics approvals from committees in each federal state and/or University Hospital for all collaborators within Germany and Austria. All methods described in this study were carried out in accordance with the.guidelines and regulations of all relevant research ethics boards. Informed consent from patients was notrequired by ethics boards due to data de-identification.

### Outcomes and definitions

Our primary outcome was an ‘adverse pregnancy event’, which included: (a) maternal-related outcomes, such as admission to ICU, need for mechanical ventilation, critical illness, or death, (b) delivery-related features, such as pregnancy termination, iatrogenic birth, surgical delivery, and stillbirth (all as a result of SARS-CoV-2 infection), and (c) neonatal-related variables, such as estimated fetal birth weight < 10^th^ centile, APGAR score at 5 min < 7, admission to the neonatal intensive care unit (NICU) due to COVID-19 (admissions to the NICU for shorter amounts of time without respiratory support while awaiting test results or for a brief period of observation were not included), and length of NICU stay greater than 24 h where there was also administration of breathing support for the neonate. Table 1 shows the variables and respective criteria used in defining adverse outcomes.

**Table 1:** Adverse pregnancy outcome composition

	**Variable**	**Criteria**
**Maternal**	Admission to adult ICU^a^	Yes
	Receiving mechanical ventilation	Yes
	Extremely critically ill^b^	Yes
	Death	Yes
**Delivery**	Termination of pregnancy due to COVID-19 associated reason	Yes
	Iatrogenic birth due to COVID-19 associated reason^c^	Yes
	Surgical delivery due to COVID-19 associated reason	Yes
	Stillbirth (birth of the infant that has died in-utero)	Yes
**Neonatal**	**Small-for-gestational-age fetus [**Estimated fetal birth weight as determined using fetal ultrasound) centile]^d^	< 10th
	Apgar score at 5-min	< 7
	Admission to NICU due to COVID-19 infection of the mother^e^	Yes
	Length of stay in NICU & receiving breathing support^f^	> 24 h & Yes

^a^ICU, intensive care unit

^b^Extremely critically ill refers to any patient for whom the clinician entering data made an assessment on whether the condition constituted a potentially life-threatening condition

^c^Iatrogenic birth has no defined delivery method, can be any delivery method as long as it is a mandatory termination of pregnancy for medical reasons and not spontaneous labour

^d^Estimated fetal birth weight was the most recent available ultrasound-estimated weight prior to the positive test

^e^NICU, neonatal intensive care unit

^f^Breathing support includes oxygen delivery through nasal cannula, high flow nasal cannula, CPAP, or intubation

### Algorithm creation

**Fig. 2:**
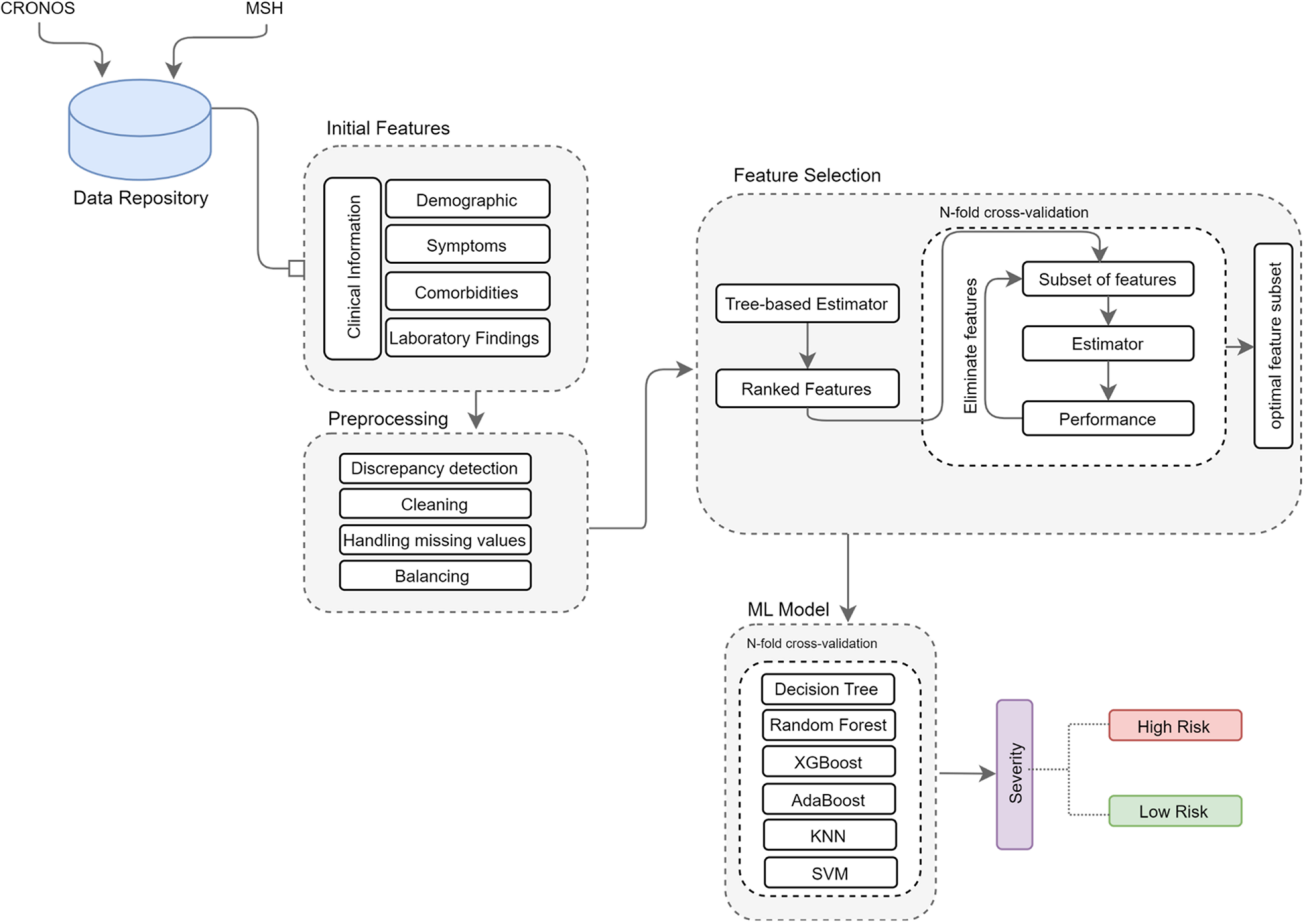
PROTECT framework

We used a binary classification task to predict the risk of adverse outcomes in SARS-CoV-2-positive pregnant patients and their neonates. The algorithm defined the possibility of an adverse outcome of a patient as either low risk or high risk. Figure 2 shows the overall architecture of our framework. Model creation consisted of three main components: preprocessing, feature selection, and model development. Full details of the algorithm creation are presented in Additional file 1: Appendix A. The following outlines the steps employed for the overall creation of the model:

1. Preprocessing
  The facilitation of the train-test process by appropriately cleaning and preparing the entire dataset. This was done by analyzing the data for discrepancies, handling missing values, scaling and balancing, and “cleaning” the data. Data cleaning primarily involved the handling of invalid values, categorizing continuous variables, and creating new features by aggregating multiple features representative of one relevant attribute.
2. Feature engineering
  The creation of new features based on knowledge of the relevant biological markers, in other words, creating composite features based on extra features that are pertinent to a single more general feature. For example, pre-existing conditions were provided as individual, binary features. These conditions were used to engineer a new feature to generate the “existence of pre-existing diseases” feature.
3. Feature selection
  The refinement of the larger feature set to generate a smaller more relevant feature subset. The most relevant features were selected based on their importance to the outcome, and achieved the greatest performance as determined through an iterative elimination process called recursive feature elimination.
4. Creation of a balanced dataset through sampling
  Addressing the imbalanced dataset by looking at class distributions of low-risk and high-risk patients in the dataset and randomly eliminating samples from the larger class. As a result, the training dataset becomes more balanced and thus allows the trained model to make more accurate, unbiased decisions.
5. Machine learning model creation
  A series of machine learning models were trained and tested using the training data from the feature selection step. These models were all evaluated separately in order to identify the best performing algorithm to be implemented in the final system.

The more complicated steps including management of missing data, feature selection, and model development are elaborated upon in the following sections. For information regarding definitions and descriptions for machine learning-related terms, refer to Table S1 of Additional file 1: Appendix A.

### Missing data

Repository data were inspected for invalid data points using the reference attribute ranges according to metadata and domain knowledge, and any invalid observations were excluded. An observation was considered invalid if all attributes for that observation had invalid values (e.g., null, 0, blank, or negatives). The data were then explored for potential discrepancies, including inconsistencies in data representation and data type, and these were manually corrected. After that exploration, the null condition was handled based on possible reasons for the missing values (e.g., in the case of the fetus, missing values for neonate and delivery attributes were filled with a new category code as “Not Applicable”).

### Feature selection

**Fig. 3:**
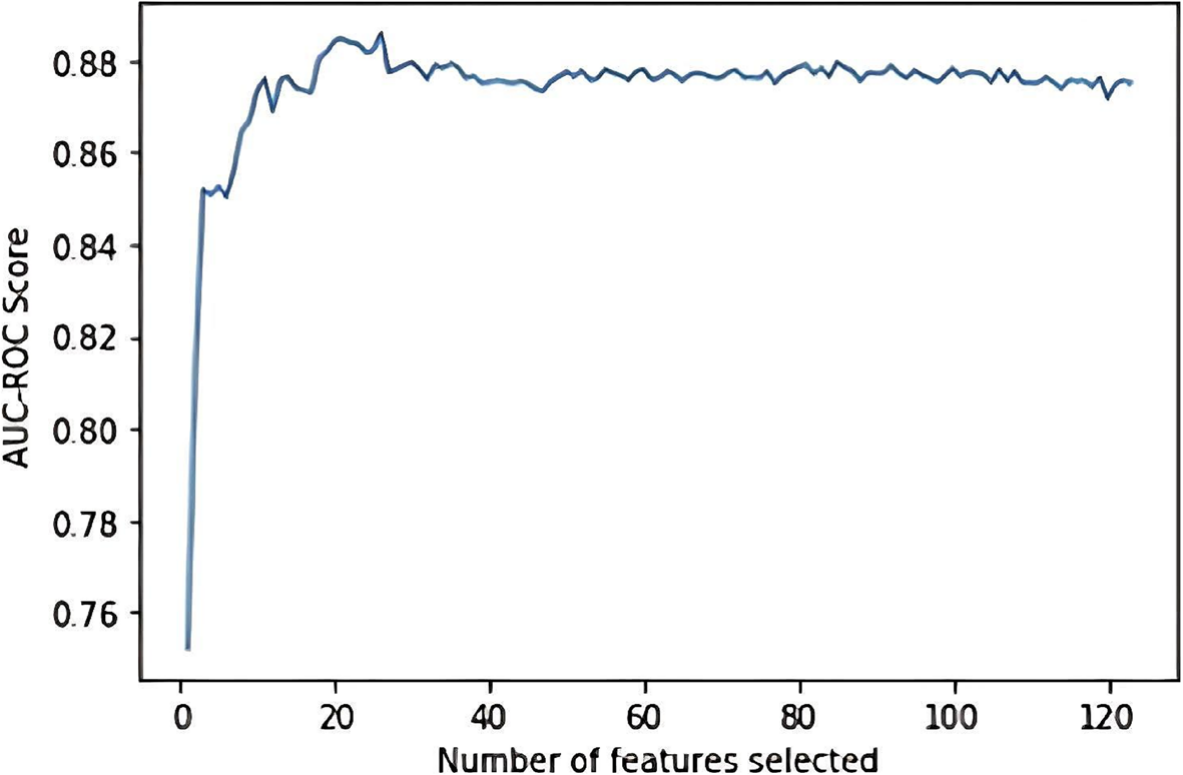
Number of features used for training versus AUC-ROC score for determining the optimal number of features using the Backward elimination approach

To identify whether an attribute impacted the endpoints considered in the feature selection phase, we fitted a random forest model with all 71 clinical attributes of the training set and calculated the model's performance using the stratified tenfold cross-validation and area under the curve (AUC) scoring. An exhaustive list of these clinical attributes prior to feature selection can be found in Additional file 1: Appendix A. Cross-validation was undergone to reduce model over-fitting. Variables were then sequentially eliminated using the wrapper-based backward elimination feature selection method (outlined in Additional file 1: Appendix A), and the model was trained on the remaining variables followed by performance calculation. The process was repeated until the most important attributes and the optimal number of features were identified based on the model’s performance. As shown in Fig. 3, a set of 25 features were selected as the optimal features to train the models. Table 2 shows the most important features to maximize the separability of classes. These features included maternal body mass index (BMI), smoking exposure, pre-existing maternal illness, duration of maternal COVID-19 symptoms (for example, dyspnea, sore throat, fever, fatigue), COVID-19 associated pneumonia, parity, gravidity, laboratory tests (for example, soluble fms-like tyrosine kinase 1 (sFLT-1) and placental growth factor (PlGF), thrombocytopenia, lactate dehydrogenase (LDH), aspartate transaminase (AST), alanine transaminase (ALT) levels), administration of antihypertensive medication, in-patient treatment due to COVID-19, maternal oxygen administration, abnormal amniotic fluid levels (assessed through fetal imaging), signs of fetal distress, presence of fetal respiratory distress syndrome (RDS) and fetal malformations, as outlined in Table 2. Continuous variables were converted to categorical ones based on low, normal, and high ranges during pregnancy.

**Table 2:** Selected features through the feature-selection phase

Category and timing when determined	Features
**Patient characteristics [Data known pre-conception or early in pregnancy]**	Maternal BMI before or at the beginning of pregnancy^a^
	Gravidity (including current pregnancy/birth)
	Parity (excluding current pregnancy/birth)
	Pre-existing diseases exist or not
**Binary outcomes (Yes/No) [Based on data obtained between conception and the positive test]**	Nicotine/smoking exposure during pregnancy
	Antihypertensive medication (at any point during pregnancy)
	Fetal malformations (structural malformation identified at 18–20 weeks of gestation)
	Did the patient experience receive antenatal corticosteroids for fetal lung maturation RDS (respiratory distress syndrome) prophylaxis at any time during this pregnancy?
**Presenting symptoms [Obtained at the time of the positive test]**	Dyspnea
	Sore throat
	Fever
	Fatigue
	Duration of symptomatic phase according to mother
**Clinical signs [Determined at the time of positive test][RD2]**	COVID-19 associated pneumonia according to clinical assessment
	Need for maternal oxygen administration
	COVID-19-associated need for inpatient treatment
**Laboratory markers [Obtained at the time of the positive test]**	Soluble fms-like tyrosine kinase 1 (sFlt-1) and placental growth factor (PlGF), ratio; sFlt-1/PlGF^b^
	Thrombocytopenia (Platelet count: < 150 × 10^9^/L)
	LDH^c^ (normal ranges between 600–800 I.U./L)
	AST or GOT^d^ (normal ranges between 200–400 U/L)
	ALT or GPT^e^ (normal ranges between 200–400 U/L)
**Assessment of fetal wellbeing [Assessed at the time of the positive test]**	Ultrasound signs of fetal distress
	Amniotic fluid (normal, polyhydramnios, oligohydramnios, or anhydramnios)

^a^*BMI* Body mass index (kg/m2)

^b^*sFlt-1/PlGF* Soluble Fms-Like Tyrosine Kinase-1/Placental Growth Factor (Preeclampsia Ratio)

^c^*LDH* Lactate dehydrogenase

^d^*AST* Aspartate aminotransferase, *GOT* Glutamic oxaloacetic transaminase

^e^*ALT* Alanine aminotransferase, *GPT* Glutamic-pyruvic transaminase

### Model development

The CRONOS and MSH datasets were used to train, validate, and test the designed framework. The models which were trained and evaluated included Support Vector Machine, k-Nearest Neighbor, Decision Tree, Random Forest, XGBoost, and AdaBoost. Both datasets were used for training to make the model more generalizable to demographics in various geographical regions. Additionally, by training on an aggregated dataset with patients from different distributions, we encourage our model to be generalizable. Furthermore, training on one dataset and validating/testing on another is an experimental setup primarily designed to evaluate AI methods specifically aimed at adapting to distribution shifts between datasets. As that task is not the focus of this paper, this approach was not used. The preprocessing step was applied to the data, after which, 262 features from the CRONOS repository and 214 features from the MSH repository were reduced to 71 features and 1501 cases. No patient records were excluded secondary to missing data. As mentioned in Additional file 1: Appendix 1, features included in the feature selection process only involved those that existed in both CRONOS and MSH repositories. These datasets included features associated with both maternal and neonatal health, thereby distinguishing the model from those created to predict COVID-19-related outcomes in the general population. Next, the Near-Miss under-sampling algorithm was used to class-balance the dataset, which resulted in selecting 562 pregnant patients with even distribution among both high and low-risk classes. Finally, the 25-feature subset obtained from the previously described feature selection phase was used to train and evaluate the six ML models. 80% of this data (562 instances with 25 features) were randomly assigned to a training and validation set and the remaining 20% was split into a test set (containing 60 high-risk patients and 52 low-risk patients).

## Results

### Study population: training and test cohorts

A total of 1501 patients were included in the aggregate data repository, with a mean (SD) age of 30.8 (5.6) years. Table 3 outlines the characteristics of the study population, separated by the existence of an adverse outcome.

**Table 3:** Repository data analysis for patients with adverse outcomes vs patients no adverse outcomes

	**Predictor Characteristics**	**CRONOS (*n* = *1402*)**		**MSH (*n* = *99*)**	
**Adverse Outcomes**	**Total participants** n (%)	***No adverse outcome***	***Adverse outcome***	***No adverse outcome***	***Adverse outcome***
		1,182 (84.3)	220 (15.7)	38 (38.4)	61 (61.6)
	**(Maternal) Admission to adult ICU** n (%)^a^	-	55 (25.0)	-	61 (100.0)
	**(Maternal) Receiving mechanical ventilation** n (%)	-	19 (9.0)	-	15 (24.6)
	**(Maternal) Extremely critically ill** n (%)	-	27 (12.3)	-	12 (19.7)
	(Maternal) Death n (%)	-	1 (0.5)	-	4 (6.6)
	**(Delivery) Termination of pregnancy due to COVID-19 associated reason** n (%)	-	31 (14.1)	-	0 (0)
	**(Delivery) Iatrogenic birth due to COVID-19 associated reason** n (%)	-	16 (7.3)	-	0 (0)
	**(Delivery) Surgical delivery due to COVID-19 associated reason** n (%)	-	27 (12.3)	-	3 (4.9)
	**(Delivery) Stillbirth** n (%)	-	25 (11.4)	-	3 (4.9)
	**(Neonatal) Estimated fetal birth weight < 10th percentile** n (%)	-	68 (30.9)	-	3 (4.9)
	**(Neonatal) Apgar score at 5 min < 7** n (%)	-	36 (16.4)	-	5 (8.2)
	**(Neonatal) Admission to NICU due to COVID-19 infection of the mother** n (%)	-	22 (10.0)	-	3 (4.9)
	**(Neonatal) Length of stay in NICU > 24 h & receiving breathing support** n (%)	-	63 (28.6)	-	1 (1.6)
**Demographics**	**Maternal age (years)** Mean (SD)	30.6 (5.5)	31.3 (6.0)	31.4 (6.3)	34.1 (4.9)
**Pregnancy Feature Analysis Outcomes**	**COVID-19-related symptom participants**^b^ n (%)	885 (74.9)	180 (81.8)	19 (19.2)	45 (73.8)
	**Pre-existing conditions**^c^ n (%)	369 (31.2)	76 (34.5)	38 (50.0)	61 (100.0)
	**Neonates tested positive for antibodies** n (%)	69 (5.8)	12 (5.5)	-	-
	**Smoking/nicotine exposure during pregnancy** n (%)	77 (6.5)	16 (7.3)	0 (0)	1 (1.6)
	**Maternal age: < 20** n (%)	23 (1.9)	6 (2.7)	0 (0)	0 (0)
	**Maternal age: 20–34** n (%)	860 (72.8)	146 (66.4)	21 (55.3)	38 (62.3)
	**Maternal age: > = 35** n (%)	275 (23.3)	66 (30.0)	15 (39.5)	23 (37.7)

^a^Given percentage is taken as a proportion of the total number of patients in that cohort, i.e. patients with adverse outcome or patients with no adverse outcome

^b^COVID-related symptoms include fever, cough, diarrhea, dyspnea, myalgia, fatigue, sore throat, malaise, nasal obstruction, headache, chest pain, altered sense of taste or smell, expectorations, and/or nausea/vomiting

^c^Pre-existing conditions include cardiovascular (i.e., hypertension), sleep apnea, preexisting diabetes mellitus, gestational diabetes mellitus, thyroid disease, metabolic disease, pulmonary disease, autoimmune disease, coagulopathy or thromboembolism, hematologic disease, gastrointestinal disease, hepatic disease, kidney disease, neurological or psychiatric illness, or any other type of preexisting condition defined by the clinician

### Model performance

**Fig. 4:**
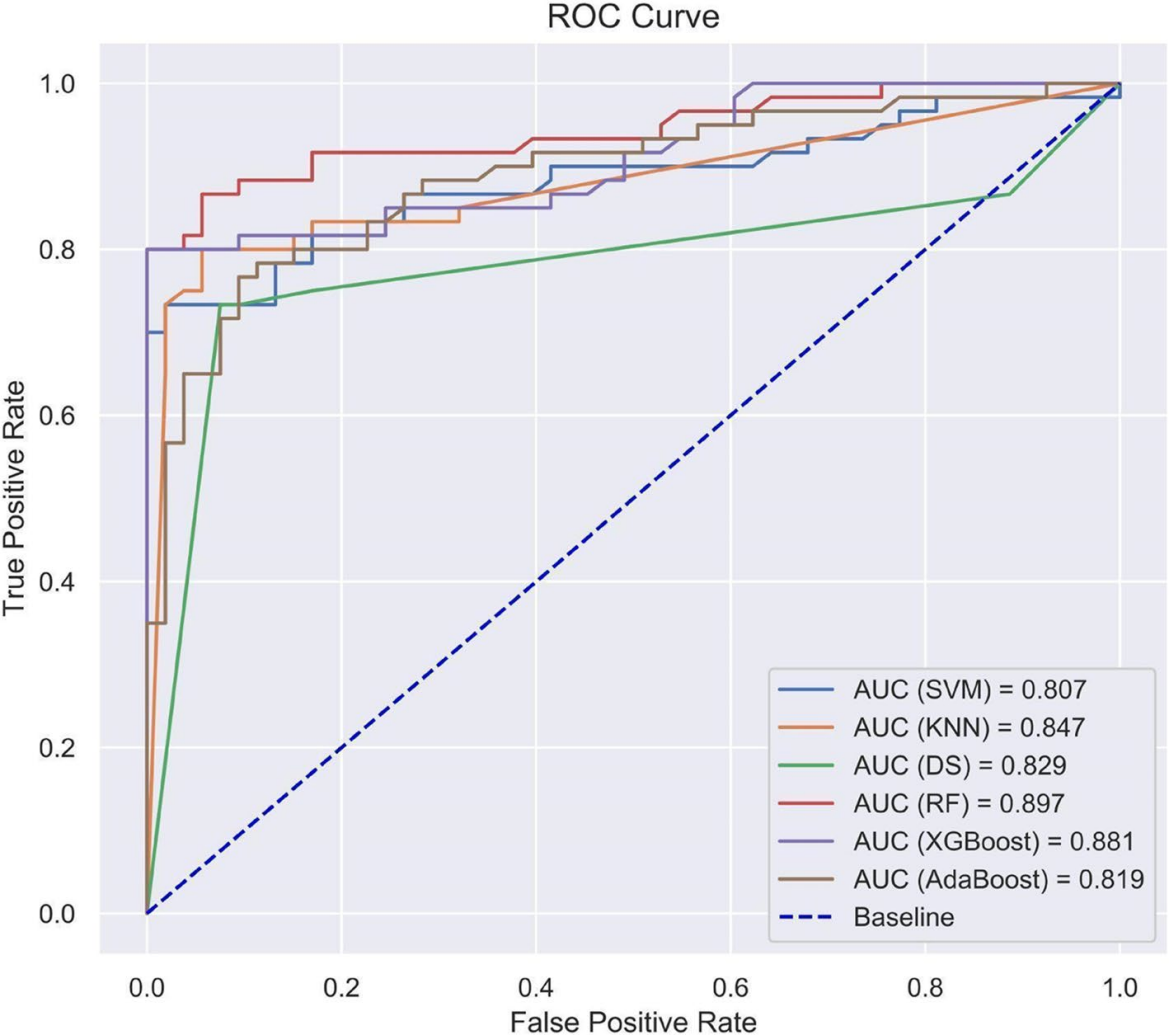
Area under ROC curve (AUC-ROC) plot for Support Vector Machine (SVM), k-Nearest Neighbors (KNN), Decision Tree (DT), Random Forest (RF), XGBoost, and AdaBoost models

**Fig. 5:**
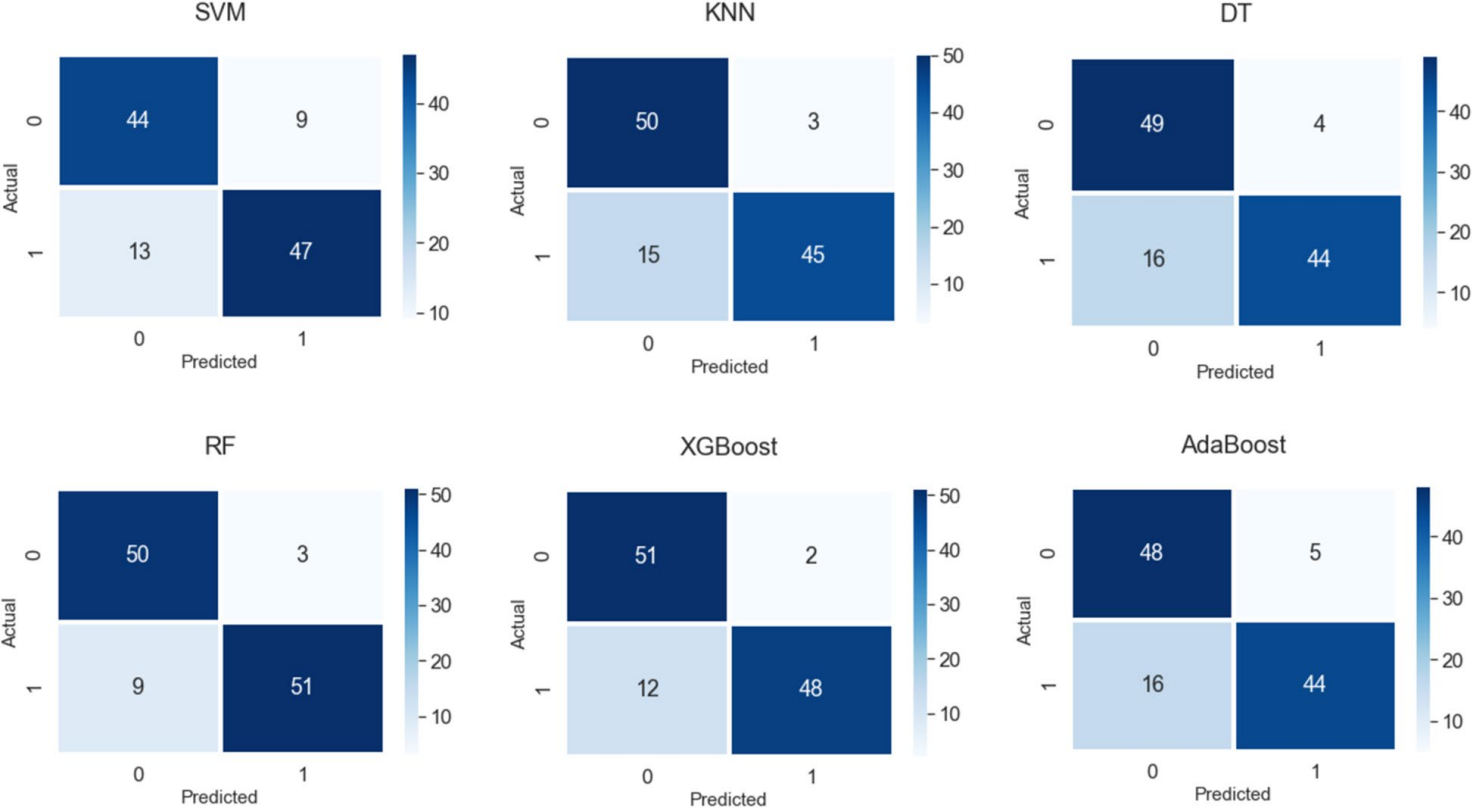
Confusion matrices of developed ML models. For each matrix, the top left box represents true negatives, top right false negatives, bottom left false positives, and bottom right true positives

As shown in Fig. 4, the ensemble Random Forest model resulted in the best overall accuracy on the test set with a score of 0.90 (95% CI 0.83 to 0.95) in predicting adverse outcomes. The test set contained 60 patients with a high risk of developing adverse outcomes and 53 patients with a low risk of developing adverse outcomes. The Random Forest model identified 51/60 (83.3%) of the high-risk patients and 50/53 (92.5%) of the low-risk patients, with an accuracy of 89.0%, a recall score of 0.85, a precision of 0.94, and an F1 score of 0.89. The performance metrics for each model are summarized in Table 4, which shows that the Random Forest model had the highest performance based on AUC, followed by XGBoost, KNN, Decision Tree, AdaBoost, and SVM. Confusion matrices for each of the developed ML models are shown in Fig. 5. Each confusion matrix in this figure demonstrates the number of correctly and incorrectly predicted adverse outcome predictions (bottom right and bottom left, respectively), and correctly and incorrectly-predicted no-adverse outcome predictions (top left and top right, respectively) that were made by each tested ML model.

**Table 4:** Model performance

	**Accuracy (%)**	**Recall**	**Precision**	**F1**	**AUC**
Support Vector Machine (SVM)	81.0	0.78	0.84	0.81	0.81 (95% CI 0.73 to 0.88)
k-Nearest Neighbour (KNN)	84.0	0.75	0.94	0.83	0.85 (95% CI 0.77 to 0.91)
Decision Tree	82.0	0.73	0.92	0.82	0.83 (95% CI 0.75 to 0.89)
Random Forest	89.0	0.85	0.94	0.89	0.90 (95% CI 0.83 to 0.95)
XGBoost	88.0	0.80	0.96	0.87	0.88 (95% CI 0.81 to 0.94)
AdaBoost	81.0	0.73	0.90	0.81	0.82 (95% CI 0.74 to 0.89)

## Conclusions

In order to improve prediction accuracy of adverse outcomes related to SARS-CoV-2 infection in pregnancy, we created an international data repository that consisted of 1501 patients from three countries. These data were then used to train and test a ML algorithm that can predict adverse maternal, fetal, and gestational outcomes with an accuracy of 89%.

While several studies have developed ML-based prognostic models for SARS-CoV-2 infection in the general population, our study is the first to present such an algorithm specifically for pregnant patients while utilizing a large SARS-CoV-2 in pregnancy data repository. This high-performance model specific to pregnancy provides a tool for clinicians to better identify at-risk pregnant patients with SARS-CoV-2 infection, allowing early hospital admission, enhanced monitoring, and proactive intervention. This algorithm has high clinical relevance as a SARS-CoV-2 infection poses significant risk to a small proportion of pregnant people, including an increased risk for critical disease requiring ventilation support as well as other maternal and fetal complications [17]. A 2020 meta-analysis including 192 international studies on the clinical manifestations, risk factors, and maternal perinatal outcomes of COVID-19 in pregnancy [2] found that the most common symptoms for pregnant or recently-pregnant women with suspected or confirmed COVID-19 were fever (40%) and cough (41%). Raised white blood cell count (26%), lymphopaenia (33%), and raised C reactive protein levels (49%) were the most common laboratory findings. They also found that pregnant women were more likely to be asymptomatic than non-pregnant women of reproductive age. Furthermore, they found that increased maternal age, high BMI, non-white ethnicity, and preexisting comorbidities were all associated with severe disease. Pregnant women with COVID-19 were found to be at increased risk of death, admission to ICU, delivering preterm, and having their neonates admitted to NICU. The determined factors related to adverse outcomes from this meta-analysis draw parallels with many of the features determined through feature selection and used to train our model. Differences include the inclusion of ethnicity as a factor to determine adverse outcome, and specific laboratory markers, such as C reactive protein and lymphopaenia. More recent studies assessing maternal vulnerability as a result of SARS-CoV-2 infection during pregnancy have consistently determined pregnant individuals to be at higher risk of hospitalization [18]. Additionally, a 2021 multinational INTERCOVID study [19] which assessed the relationship between maternal factors such as gestational diabetes mellitus, diabetes mellitus, and maternal obesity related to COVID-19 diagnosis. Interestingly, it was found that these conditions were associated with higher risk of COVID-19 disease severity, both of which were included within the features selected to train our prognostication model.

Our algorithm significantly improves upon previously published AI techniques for COVID-19 prognosis. For example, Schöning et al [20] created a clinical score and ML model that predicted the likelihood of severe disease courses from SARS-CoV-2, where a severe outcome was defined as admission to ICU and/or death. The measured metrics in the validation cohort included an AUC of 0.85, positive predictive value (PPV) of 0.91, and negative predictive value (NPV) of 0.81. However, this study was limited by poor censoring of outcomes (given there was no explicit follow-up), a small sample size (*n* = 657) and no laboratory data available for included subjects. Similarly, Rechtman et al [21] developed an XGBoost model for the prediction of COVID-19 mortality using 8770 confirmed SARS-CoV-2 cases, including demographic, clinical, and comorbidity variables taken from all Mount Sinai Health System facilities in New York, USA. Cross-validation results yielded an AUC of 0.86 when combining effective predictors. However, this study was limited by poor generalizability due to the absence of widespread testing at the time of algorithm development, with only severe cases of COVID-19 included in the model. Moreover, there was limited access to ICU admission and oxygen support information, thereby limiting the number of patients with severe outcomes that were included in the model. Our algorithm leverages a large, varied dataset with detailed follow-up results and obtains competitive performance or outperforms the existing systems. By using a Random Forest model, a type of Ensemble Learning approach, we were able to overcome the challenges of high disease course variability to accomplish high COVID-19 prognostication accuracy [22]. This problem of generalizability is common in studies attempting to create mortality prediction algorithms [21, 23, 24] and is often caused by data sparsity, leading to a lack of validation from external healthcare systems [25]. The use of Random Forest for the wrapper-based feature selection technique allows for better model building with a large number of clinical features, however also serves as a limitation due to the fact that using the same algorithm at both feature selection and learning stages can inflate performance results [26]. Although studies may find promising performance results in the training phase using COVID-19 repositories, this does not guarantee the generalizability of the algorithm. As such, including diverse care settings or geographic regions can considerably improve the generalizability of the algorithm [27]. Therefore, our study set out to retrieve data from separate sources of different geographic locations to allow for evaluation on more diverse representative data. Of these sources, the CRONOS repository contained data from centers throughout Germany and Austria, thereby furthering the geographical variability of our repository. The large size and diversity of our international repository allowed for the development of a trained ensemble algorithm capable of providing accurate outcomes using a combination of data input parameters.

Nevertheless, this study has several limitations. Firstly, the repository included patient data only for individuals who either sought medical care because of SARS-CoV-2 infection or were found positive on testing prior to giving birth. Our data may not be a real reflection of the general population of pregnant women affected by COVID-19, since health and lifestyle factors may differ in demographics without access to medical care. Canada, Germany, and Austria are predominantly white countries, which may result in the presence of racial bias in the model [28] Therefore, we do not recommend widespread use of this algorithm before future advancements allow for it to be validated in diverse populations. Future advancements of this model aim to diversify and expand the patient demographics in newly obtained data, including racial diversification. Next, as shown in Table 3, the percentage of participants with adverse outcomes differed between the two data sources. There could be several reasons for differences in outcomes between the two datasets. First, CRONOS is a large registry with a broad range of patients from Germany and Austria. Mount Sinai Hospital is a tertiary level hospital in Toronto, Canada which serves as a referral centre for the Greater Toronto Area. The patient population here is a higher-risk population than in the CRONOS registry. The protocols for each registry differ greatly, as CRONOS is a prospective database from over 100 sites, including those admitted and those not requiring admission. MSH, on the other hand, is a list of very sick people admitted to the hospital, not necessarily including everyone who screened positive. CRONOS was a universal screening, whereas MSH was a symptom-based screening and, therefore, was not a comprehensive list of everyone that may have tested positive. By including these vastly different data sources, we were able to obtain a good balance between “population-based” and “hospital-based” screening, thus providing a rich dataset of cases with varying severity to inform the model. As such, our algorithm is based on data obtained from both a high and a low-risk population. Furthermore, one of the adverse outcome features used was admission of the neonate to the NICU due to maternal SARS-CoV-2 infection. However, the reasoning for this admission may vary across different hospitals and health centers, depending on local regulations. For example, NICU admission may be cautionary only at some centres, while in others it may be due to a medical need. Such variable definition limits the accuracy of the model, which could potentially incorrectly-label patients with adverse outcomes, depending on one’s interpretation. Similarly, another variable defining adverse outcome is described as the patient is “extremely critically ill”. This variable is also subject to inter-subject variability and differing interpretations, as there is no objective or quantifiable threshold to help define it. It should also be noted that, as with most machine learning models, the exclusion of certain feature inputs when calculating a patient’s risk may result in a deterioration of the model’s performance. Therefore, collection of all input features (as outlined in the Study and Population section) should be undertaken to ensure maximal model performance. The features we utilise with this model are routine in obstetrics and, thus, should be readily available in most non-emergency cases, making our algorithm usable in a typical pregnancy. Our algorithm is not intended to be used in emergency cases, where these features are most likely to be missing, as the emergency will merit immediate medical intervention regardless of the predicted future risk.

The absence of vaccination status as an input feature to the model serves as an additional limitation. mRNA vaccination has shown to be effective and safe for reducing the severity of COVID-19 in pregnancy [29]. Recent observational studies have evaluated COVID-19 vaccine effectiveness in pregnant patients during the first 6 months of omicron as the variant of concern [30]. Administration of complete or boosted vaccines were found to reduce the risk for severe symptoms, complications, and death [30]. Another 2023 study determined vaccination to be highly effective against delta variants when infected during gestation, and moderately effective against omicron [31]. This being said, the inclusion of vaccination status as a feature for model training using more recent patient data would likely be indispensable for the model, and serve as an important factor in prognostication. Unfortunately, the data collected for the training of this model was done so prior to the availability of any vaccines. Additional data were provided for the CRONOS repository later in the course of this project, which included 1013 patients with known vaccination status. This vaccine variable contained ambiguity as to whether the patients received one or multiple doses. After this additional data were preprocessed, the vaccination feature was deemed unnecessary by the feature selection algorithm and thus was eliminated. The ambiguity of the feature may attest to its elimination, and provision of a more concise feature (indicating number of doses received) may be more impactful for our future model advancements. Therefore, the inclusion of vaccination status as an input feature for this prognostication model does not necessarily allow for a higher-accuracy model. However, future studies will attempt to advance this model by expanding the data repository to include populations from broader global regions. The expanded data repository will also aim at acquiring vaccination status as a multi-class variable reflecting the number of doses received. With this improved dataset, the vaccination feature may have a greater impact on adverse outcome prediction. While we attempted to increase the heterogeneity of our dataset by including patient data from three different countries, we hope to train our algorithm with data from additional countries to further broaden the generalizability of our algorithm. Ultimately, we plan to develop an accessible user interface platform for healthcare providers and patients to input relevant pregnancy parameters that will provide a meaningful prediction of risk.

In conclusion, we demonstrated that clinical data can effectively predict disease severity in pregnant patients with SARS-CoV-2 infection. The intention is to use the algorithm at the point of a first positive test. However, knowing that risk is dynamic, the algorithm should be used each time the clinical condition changes, for e.g., if there are worsening respiratory symptoms or a new pregnancy-related clinical finding. Implementation of this platform and continued validation through international use could significantly improve the ability of healthcare providers to identify, manage, and treat the disease in pregnant people worldwide.

## Supplementary Information

**Additional file 1:** **Appendix A.** (Methods). **Table S1.** Machine learning term definitions. **Table S2.** Full feature list prior to selection of feature subset using gridsearch [32–46].

## Data Availability

The data repository generated and used in this study is not publicly available due to the collaborating hospitals’ restrictions on data sharing. However, a summary of the CRONOS data is regularly updated and is available here: https://www.dgpm-online.org/gesellschaft/covid-19/. Additionally, the trained algorithm is available on Github:https://github.com/MFI-Lab/PROTECT. For further queries, please contact the corresponding author.

## Abbreviations

COVID-19: Coronavirus disease of 2019
SARS-CoV-2: Severe acute respiratory syndrome coronavirus 2
ARDS: Acute respiratory distress syndrome
ICU: Intensive care unit
AI: Artificial intelligence
ML: Machine learning
TRIPOD: Transparent reporting of a multivariable prediction model for individual prognosis or diagnosis
CRONOS: COVID-19 related obstetric and neonatal outcome study
DGPM: German society of perinatal medicine
MSH: Mount Sinai hospital
NICU: Neonatal intensive care unit
AUC: Area under the curve
BMI: Body mass index
sFLT-1: Soluble fms-like tyrosine kinase 1
PlGF: Placental growth factor
LDH: Lactate dehydrogenase
AST: Aspartate transaminase
ALT: Alanine transaminase
RDS: Respiratory distress syndrome
SD: Standard deviation
KNN: K-Nearest neighbour
RF: Random forest
DT: Decision tree
COVID-19: Support vector machine
PPV: Positive predictive value
NPV: Negative predictive value
