# Supplementary material for: Predicting adverse outcomes in pregnant patients positive for SARS-CoV-2: a machine learning approach- a retrospective cohort study
Source: BMC Pregnancy Childbirth. 2023 Aug 2;23:553. doi: 10.1186/s12884-023-05679-2 (PMC10394879; doi:10.1186/s12884-023-05679-2)
Supplement: Supplementary file 1 — Additional file 1: Appendix A. (Methods). Table S1. Machine learning term definitions. Table S2. Full feature list prior to selection of feature subset using gridsearch [32–46]. [file 12884_2023_5679_MOESM1_ESM.docx]

**Predicting adverse outcomes in pregnant patients positive for SARS-CoV-2: a machine learning approach- a retrospective cohort study**

**Appendix A (Methods)**

### **Preprocessing**

Data preprocessing is the prerequisite and an important step for ML model development.3^2-34^ This step aims to facilitate the train-test process by appropriately cleaning, preparing, and transforming the entire dataset. We adopted a multi-step approach for preprocessing, including discrepancy detection, cleaning, handling missing values, scaling and balancing, utilizing information from the data, metadata, and domain knowledge.

Data were inspected regarding invalid observations and values, using the reference attribute ranges according to the metadata and domain knowledge, followed by dropping invalid observations. Specifically, an observation was considered invalid if all attributes for that observation have invalid values (e.g., null, 0, blank and negatives). Then, data were explored for potential discrepancies including data representation and data type inconsistencies, and the issue was addressed based on properties of the data and domain knowledge (e.g., in the case where there was an instance with explicit indication of the patient being “asymptomatic”, but instance symptom/symptoms were specified, the related attribute was overwritten to indicate a symptomatic patient). After that exploration, null condition was handled based on possible reasons for having missing values (e.g., in case of unborn child, missing values for neonate and delivery attributes were filled with a new category code as “Not Applicable”). Additionally, after the data had been cleaned, new attributes were created using the existing ones to aggregate similar correlated attributes into one (e.g., smoking habits which originally represented 3 attributes, aggregated into one, representing whether a participant smoked or was exposed to nicotine pre/during pregnancy).

Finally, as the data were skewed towards the low-risk class with the ratio of (1:14) 0.14, this class imbalance could negatively affect the ability of a classifier to make accurate predictions, regardless of the applied learning method.^35,36^ As such, we adopted the Near-Miss-2 under-sampling approach^37^ to address the imbalanced distribution of patients across the decision classes. We calculated the average distance to the minority instances and selected instances from the majority class with the smallest average distance to the three furthest instances from the minority class. Undersampling (rebalancing) was done to enable the model during training to learn about not just the majority class, but also the minority class. Other metrics including precision, recall, and f1 measure are also reported. ML models are evaluated in this manner to avoid selecting models that simply overfit to whatever population bias they were trained on. We believe that the extensive metrics we present prove our model's utility and capability for correctly identifying at-risk patients, despite the synthetic balanced sample it was tested on.

### **Feature Selection**

Recursive feature elimination^38^ was applied as a hybrid method for feature selection to acquire ranking of features through the use of a tree-based estimator. A wrapper method was established using a backward elimination to search for the best possible feature subset.^38^ In other words, a random forest estimator was first trained on the initial set of features, and the importance of each feature was obtained. Subsequently, the least important features were pruned from the current set of features. That procedure was recursively repeated on the pruned set until the optimal number of features was obtained using cross-validation. To elucidate this process, this estimator determines feature importances by first calculating the feature importances of a node j in a single decision tree (assuming only two child nodes) by:

${ni}_{j}=w_{j}C_{j}-w_{left(j)}C_{left(j)}-w_{right(j)}C_{right(j)}$ (1)

Where ${ni}_{j}$ is the importance of node j, $w_{j}$ is the weighted number of samples in node j as a fraction of the total weighted number of samples, $C_{j}$ is the impurity node in j, and left(j) and right(j) are its respective children nodes^39^. Therefore, the importance, (fi) for each feature (i) is then calculated using:

${fi}_{i}=\frac{\sum_{j: node j splits on feature i}{ni}_{j}}{\sum_{k\epsilon all nodes}{ni}_{k}}$ (2)

where ${fi}_{i}$ is the importance of feature i and ${ni}_{j}$ is the node importance as calculated using equation (1).

The resultant feature subset was used to train each tested model, rather than computing a new feature subset using an estimator related to the model being trained. This was likely a reason why the R

andom Forest model yielded the highest performance, as it has been found that when models are trained using the same learning algorithm as in the feature selection stage, the subsequent prediction task using the features selected by the wrapper achieves better performance.^26^

### **Model Development**

For the adverse outcomes considered, a series of classifiers including Support Vector Machine (SVM),^40^ K-Nearest Neighbor (KNN),^41^ Decision Tree (DT),4^2^ Random Forest (RF),4^3^ Extreme Gradient Boosting (XGBoost)4^4,45^ and Adaptive Boosting (AdaBoost),^46^ were trained using the selected attributes from the previous step as predictors to classify patients as higher or lower risk in terms of developing the adverse outcomes.

*Statistical analysis*

Exploratory data analysis was conducted throughout the process of data preprocessing to aid in algorithm development. This allowed for the determination of the sparsity of instances for each feature provided and the reformatting and imputation of data features for ML model training. The CRONOS repository was retrieved first, as it was equipped with more features and attributes (283 features with 1402 instances and 214 features with 99 instances in CRONOS and MSH datasets, respectively) than the MSH repository. This prompted the resulting imputation and feature selection methodologies to revolve around only features that were present in both datasets. Thus, imputation of features and engineering of new features based on existing features was undertaken using the CRONOS repository as a reference. Exploratory data analysis of individual repositories stratified by adverse outcomes is shown in Tables 2 and 3. Although the MSH repository contained fewer features than the CRONOS repository (*n*=214), determinant features of adverse outcomes were all present in the MSH data.

Lab result features were categorized based on research findings for normal, high, and low levels specific to pregnant individuals of each respective attribute. This included both laboratory findings (i.e., Haemoglobin, AST) and other continuous variables such as Gravida and Apgar scores.

*Outcomes for Training and Testing*

We used the selected features as an input in the model development phase and trained 6 different predictive models using the training set. Each model was separately optimized by 10-fold cross validation grid search, wherein the models were exhaustively trained and cross-validated over a grid of possible parameters including number of estimators, number of features considered at every split, number of levels in tree, minimum number of samples required to split a node, minimum number of samples required at each leaf node, and whether or not there is bootstrapping for sample selection in each tree performance of each selected model was then evaluated on the test set. The complete list of features used for this grid search can be found in Table S2 below. Hyperparameters of the 6 models were as follows: (1) the SVM model with radial basis function kernel, gamma set to 1, L2 regularization penalty set to 0.1; (2) the KNN model with 3-nearest neighbors’ vote, leaf-size of 30, and considering weight points by the inverse of their Euclidean distance; (3) the decision tree model used a Gini impurity function to measure the quality of split (to help identify which features best classify the target outcome), with maximum depth of the tree set to 12, and minimum sample split set to 2; (4) the Random Forest model with bootstrapping was trained using 500 trees, splits using Gini impurity, maximum tree depth of 20, and minimum samples split of 5; (5) the XGBoost model was trained with its hyperparameters set to 1000 estimators, maximum tree depth of 15, the learning rate of 0.5, subsampling occurring once in every boosting iteration, and L2 regularization to minimize log-loss; (6) the AdaBoost was trained with 500 decision trees as its weak estimators, the learning rate of 0.01, and minimum samples split of 2.

#

# Tables

*Table S1. Machine learning term definitions*

| **Term** | **Definition** |
| --- | --- |
| ***Feature*** | An individual independent variable acting as inputs to the algorithm (e.g. presence of a cough) |
| ***Instance*** | An example in the training data (e.g. a single patient) |
| ***Attribute*** | A parameter which may or may not be a feature |
| ***Target*** | A feature (see above) used for the predictive training of a model, which is predicted by other features (e.g. adverse outcomes, such as admission to ICU due to COVID-19) |
| ***Artificial Intelligence*** | Computers and machine programs that mimic the human mind’s decision-making and problem-solving capabilities |
| ***Machine Learning*** | A type of artificial intelligence where computer algorithms are designed to improve automatically through experience and using data to perform certain tasks |
| ***Support Vector Machine*** | A type of algorithm that creates lines (also known as hyperplanes) to separate data into classes |
| ***k-Nearest Neighbor*** | A type of algorithm that classifies data based on a similarity measure calculated from distance functions |
| ***Decision Tree*** | Type of algorithm that takes input data and recursively partitions it so to improve the “purity score” of a sub-table to identify certain classes |
| ***Random Forest*** | Type of (ensemble) algorithm consisting of a large number of decision trees operating as an ensemble |
| ***XGBoost*** | Type of (ensemble) algorithm consisting of gradient-boosted decision trees applying the principle of boosted weak learners |
| ***AdaBoost*** | Type of (ensemble) algorithm that also applies the boosting technique and re-assigns weights to each instance, with higher weights to incorrectly classified instances |
| ***Wrapper Method*** | A subset of features is used to train and test the model. Inferences from the previously-trained model are used to add or remove features from the subset for re-training |
| ***Backward Elimination*** | All features are used to initially train the model, with the least significant features removed to iteratively re-train the model for improved performance |

*Table S2. Full feature list prior to selection of feature subset using grid search.*

| *Category and timing when determined* | *Features* |
| --- | --- |
| *Patient characteristics*  *[Data known pre-conception or early in pregnancy]* | *Maternal BMI before or at the beginning of pregnancy^^[[1]](#footnote-1)^^* |
|  | *Gravidity (including current pregnancy/birth)* |
|  | *Parity (excluding current pregnancy/birth)* |
|  | *Pre-existing diseases exist or not* |
|  | *Maternal age* |
| *Binary outcomes (Yes/No)*  *[Based on data obtained between conception and the positive test]* | *Nicotine/smoking exposure during pregnancy* |
|  | *Nicotine/smoking exposure before pregnancy* |
|  | *Nicotine/smoking exposure in the home/because of partner* |
|  | *Antihypertensive medication (at any point during pregnancy)* |
|  | *Current other medications of the mother, including:*  *None, antibiotics, aspirin (100 to 150 mg), asthmatic medication, antidiabetic medication, anticoagulants, or other (1 feature for each, 7 features total)* |
|  | *Asthma medications other than glucocorticoids or betamimetics* |
|  | *Iatrogenic termination of pregnancy* |
|  | *Serious obstetrical indication, i.e. FGR* |
|  | *Mode of delivery, including: natural birth, vaginal birth, schedule C-section, unplanned C-section, vaginal birth after C-section, or unknown/* |
|  | *Was the anesthesiologist involved in the treatment support?* |
|  | *Fetal malformations (structural malformation identified at 18-20 weeks of gestation)* |
|  | *Was the fetal malformation of the child known prenatally?* |
|  | *Was the fetal malformation of the child known prior to the COVID-19 infection?* |
|  | *Did the patient experience receive antenatal corticosteroids for fetal lung maturation RDS (respiratory distress syndrome) prophylaxis at any time during this pregnancy?* |
|  | *Which drug was used for induction of lung maturity? (If needed): Betamethasone, Dexamethasone, or other.* |
|  | *Index contact known? Family member positive or with the same symptoms?* |
|  | *Multiple pregnancies* |
|  | *Nicotine/smoking exposure before pregnancy* |
|  | *Nicotine/smoking exposure in the home/because of partner* |
|  | *Fetal birth weight* |
|  | *Chorionicity* |
| *Presenting symptoms*  *[Obtained at the time of the positive test]* | *Dyspnea* |
|  | *Sore throat* |
|  | *Fever* |
|  | *Fatigue* |
|  | *Duration of symptomatic phase according to mother* |
|  | *Any symptoms consistent with the COVID-19 infection* |
|  | *Cough* |
|  | *Diarrhea* |
|  | *Expectorations/sputum ejections* |
|  | *Myalgia* |
|  | *Malaise* |
|  | *Nasal breathing obstructions* |
|  | *Thorax/chest pain* |
|  | *Headache* |
|  | *Dizziness or light-headedness* |
|  | *Altered sense of smell or taste* |
|  | *Nausea or vomiting* |
|  | *Other symptoms* |
| *Clinical signs*  *[Determined at the time of positive test][RD2]* | *COVID-19 associated pneumonia according to clinical assessment* |
|  | *Need for maternal oxygen administration* |
|  | *COVID-19-associated need for inpatient treatment* |
|  | *Outpatient management* |
| *Laboratory markers*  *[Obtained at the time of the positive test]* | *Soluble fms-like tyrosine kinase 1 (sFlt-1) and placental growth factor (PlGF), ratio; sFlt-1/PlGF^^[[2]](#footnote-2)^^* |
|  | *Thrombocytopenia (Platelet count: < 150 × 109/L)* |
|  | *LDH^^[[3]](#footnote-3)^^ (normal ranges between 600-800 I.U./L)* |
|  | *AST or GOT^^[[4]](#footnote-4)^^ (normal ranges between 200-400 U/L)* |
|  | *ALT or GPT^^[[5]](#footnote-5)^^ (normal ranges between 200-400 U/L)* |
|  | *Haemoglobin (normal ranges between 12-16 g/dL)* |
|  | *Haptoglobin (normal ranges between 50-220 mg/dL)* |
| *Assessment of fetal wellbeing*  *[Assessed at the time of the positive test]* | *Ultrasound signs of fetal distress (assessed via Doppler ultrasound)* |
|  | *Amniotic fluid (normal, polyhydramnios, oligohydramnios, or anhydramnios)* |
|  | *Suspected preterm birth; preterm contractions* |
|  | *Premature rupture of membranes* |
|  | *Cervical insufficiency* |
|  | *Fetal growth restriction* |
|  | *Abnormal fetal heart rate* |
|  | *Hypertensive disorders in pregnancy* |
|  | *HELLP syndrome* |
|  | *Intrahepatic cholestasis of pregnancy* |
|  | *Other reason for admission* |
|  | *Is the child COVID-19 antibody positive?* |
|  | *Was a COVID-19 smear PCR test in the child positive?* |

1. BMI, body mass index (kg/m^2^) [↑](#footnote-ref-1)
2. sFlt-1/PlGF, Soluble Fms-Like Tyrosine Kinase-1/Placental Growth Factor (Preeclampsia Ratio) [↑](#footnote-ref-2)
3. LDH, lactate dehydrogenase [↑](#footnote-ref-3)
4. AST, aspartate aminotransferase, GOT, glutamic oxaloacetic transaminase [↑](#footnote-ref-4)
5. ALT, alanine aminotransferase, GPT, glutamic-pyruvic transaminase [↑](#footnote-ref-5)
